# Assessment of patient doses during mammography practice at Kenyatta National Hospital

**DOI:** 10.1186/bcr2996

**Published:** 2011-11-04

**Authors:** JS Wambani, GK Korir, MN Shyanguya, IK Korir

**Affiliations:** 1 Kenyatta National Hospital, Nairobi, Kenya; 2 University of Massachusetts Lowell, MA, USA; 3 National Nuclear Regulator, Johannesburg, South Africa

## Introduction

Breast cancer constitutes 21% of all cancer cases and ranks third according to cancer type in Kenya. Mammography therefore remains a powerful radiographic imaging technique for detecting and managing breast cancer. However, the active and radiosensitive glandular tissue calls for the need for an effective quality assurance programme.

## Methods

A questionnaire method was developed and used in recording the displayed patient dose, compressed breast thickness and exposure factors. The average glandular dose, device performance, and film quality grading were also carried out at the largest mammography facility in Kenya.

## Results

There were 3,264 films from 1,252 women of between 25 and 90 years old. The AGD per film was 2.14 (range 0.27 to 9.43) mGy for the CC projection and 2.44 (range 0.20 to 10.12) mGy for the MLO projection. A total 17% of CC and 30% of MLO films recorded doses above the recommended 3 mGy diagnostic reference level.

## Conclusion

In view of the diagnostic dose finding of this study, the variation of mammography imaging techniques revealed the need for standards and optimisation of mammography practice in Kenya.

